# A Dual Molecular Biointerface Combining RGD and KRSR Sequences Improves Osteoblastic Functions by Synergizing Integrin and Cell-Membrane Proteoglycan Binding

**DOI:** 10.3390/ijms20061429

**Published:** 2019-03-21

**Authors:** Mireia Hoyos-Nogués, Elena Falgueras-Batlle, Maria-Pau Ginebra, José María Manero, Javier Gil, Carlos Mas-Moruno

**Affiliations:** 1 Biomaterials, Biomechanics and Tissue Engineering Group (BBT), Department of Materials Science and Metallurgical Engineering, Universitat Politècnica de Catalunya (UPC), 08019 Barcelona, Spain; mhoyos@uic.es (M.H.-N.); elena.falgueras9@gmail.com (E.F.-B.); maria.pau.ginebra@upc.edu (M.-P.G.); jose.maria.manero@upc.edu (J.M.M.); xavier.gil@uic.cat (J.G.); 2 Barcelona Research Center in Multiscale Science and Engineering, UPC, 08019 Barcelona, Spain; 3 Universitat Internacional de Catalunya (UIC), 08195 Sant Cugat del Vallès, Spain

**Keywords:** biointerface, titanium, functionalization, RGD, integrin, KRSR, proteoglycan, osteointegration, osteoblast, coating

## Abstract

Synergizing integrin and cell-membrane heparan sulfate proteoglycan signaling on biomaterials through peptidic sequences is known to have beneficial effects in the attachment and behavior of osteoblasts; however, controlling the exact amount and ratio of peptides tethered on a surface is challenging. Here, we present a dual molecular-based biointerface combining integrin (RGD) and heparin (KRSR)-binding peptides in a chemically controlled fashion. To this end, a tailor-made synthetic platform (PLATF) was designed and synthesized by solid-phase methodologies. The PLATF and the control linear peptides (RGD or KRSR) were covalently bound to titanium via silanization. Physicochemical characterization by means of contact angle, Raman spectroscopy and XPS proved the successful and stable grafting of the molecules. The biological potential of the biointerfaces was measured with osteoblastic (Saos-2) cells both at short and long incubation periods. Biomolecule grafting (either the PLATF, RGD or KRSR) statistically improved (*p* < 0.05) cell attachment, spreading, proliferation and mineralization, compared to control titanium. Moreover, the molecular PLATF biointerface synergistically enhanced mineralization (*p* < 0.05) of Saos-2 cells compared to RGD or KRSR alone. These results indicate that dual-function coatings may serve to improve the bioactivity of medical implants by mimicking synergistic receptor binding.

## 1. Introduction

One of the main concerns in the field of implantology is to improve the biointegration of the implanted materials with surrounding tissues. Indeed, incomplete osteointegration stands out as a major source of implant failure [1,2]. To this end, biomolecular coatings aiming at stimulating and enhancing cell responses have been reported [3,4,5,6]. This has been achieved by modifying the biomaterial surface with receptor-binding organic molecules that mimic the extracellular matrix (ECM) of bone [7,8,9,10]. Although the selection of an adequate coating molecule is still a matter of discussion [11], it is well accepted that synthetic peptides offer advantages in terms of reproducibility, handling and stability compared with native ECM proteins. However, single peptide motifs are also limited, often showing less biological potential and poor selectivity profiles [10,12]. To overcome these drawbacks, a useful approach is to install multifunctionality on surfaces by combining distinct bioactive sequences having complementary or synergistic biological effects [13,14,15].

For example, the well-known cell adhesive Arg-Gly-Asp (RGD) motif enhances osteoblastic functions by binding to integrins expressed on bone forming cells, e.g., αvβ3 and α5β1 [10,16]. The RGD sequence, nonetheless, is also able to bind to other integrins [17] and may thus promote nonspecific cell adhesion. A feasible approach to selectively improve osteoblastic responses is to use peptides with affinity for cell surface heparan sulfate proteoglycans, such as Lys–Arg–Ser–Arg (KRSR) and Phe-His-Arg-Arg-Ile-Lys-Ala (FHRRIKA). More specifically, KRSR is a heparin-binding peptide designed following a consensus pattern found in five different bone-related adhesive proteins (fibronectin, vitronectin, bone sialoprotein, thrombospondin, and osteopontin), which is known to specifically promote adhesion of osteoblasts and osteogenic differentiation [18,19,20,21,22,23]. 

Hence, incorporating both integrin- and heparin-binding domains on biomaterial surfaces has potential to enhance the implant’s bioactivity and the formation of mineralized tissue, by stimulating an effective crosstalk between integrins and cell-membrane proteoglycans. Indeed, early studies by Rezania and Healy showed improved osteoblast adhesion and mineralization when combining RGD with FHRRIKA on titanium surfaces [24]. More recently, Beck-Sickinger and co-workers reported enhanced osteoblastic responses in a cooperative manner presenting a cyclic RGD peptide and FHRRIKA within the same molecule [25]. The combination of RGD and KRSR on fibrillar hydrogels also resulted in positive synergistic effects on osteoblast-like cells [26]. However, other studies reported rather modest or even negative results. For instance, Bell et al. described that the co-presentation of RGD and KRSR inhibited osteoblast differentiation [27], and the same peptides failed to promote new bone formation in an in vivo study [28]. In another study, the combination of these two sequences did not provide any evidence for synergistic effects [29].

The lack of activity observed in these studies was attributed to the random presentation of the peptide motifs on the surfaces, which probably could not reproduce the optimal distance and orientation required for an effective signaling. This is a common limitation associated with the use of peptide mixtures [12,30]. As a matter of fact, the geometrical disposition and proportion of integrin- and heparin-binding peptides are critical factors strongly affecting the biological outcome [20,25,31]. Thus, the non-homogenous distribution of the peptides is expected to render inconsistent and variable results, and would partially explain the discrepancies observed in the literature.

In response to these limitations, we previously developed a synthetic double-branched peptidic structure for the presentation of bioactive sequences in a chemically defined fashion [30]. In detail, this molecule was used to synergize the binding to α5β1 on titanium by mimicking the geometrical spacing of RGD and Pro-His-Ser-Arg-Asn (PHSRN) present in fibronectin [30,32]. Interestingly, this system of presentation outperformed the linear peptides and their combination using random peptide mixtures. In subsequent works, the same type of molecule was used to combine cell adhesive and antimicrobial peptides [33,34].

**Figure 1:**
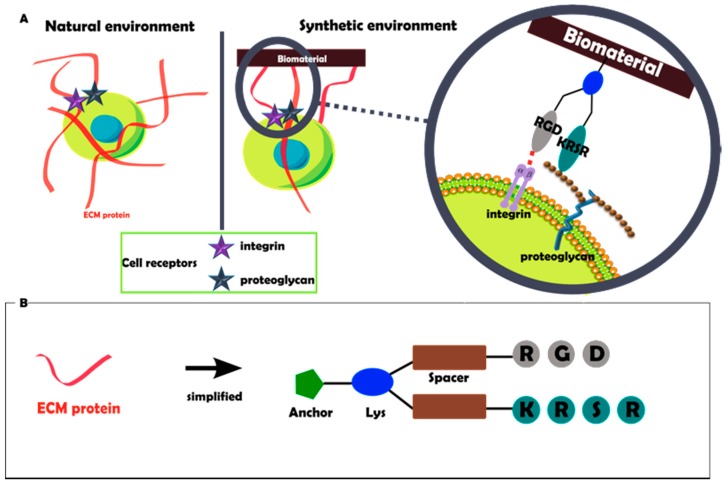
(**A**) Native ECM proteins found in bone tissues present integrin and cell-membrane heparan sulfate proteoglycan-binding ligands to stimulate and control cell behavior. Such biofunctional microenvironment can be synthetically replicated by means of designing molecular interfaces co-presenting integrin (RGD) and proteoglycan (KRSR)-binding peptides. (**B**) Schematic representation of the synthetic platform (PLATF), a minimalistic approach to recapitulate ECM multifunctionality. The molecule contains suitable anchoring, branching (Lys) and spacing units, and the bioactive sequences RGD and KRSR.

Therefore, the aim of the present work was to develop a synthetic dual biomolecular interface, capable of mimicking the natural ECM microenvironment and synergistically interacting with integrin and proteoglycan cell receptors (Figure 1A). For this purpose, we introduce the use of a tailor-made synthetic platform (PLATF) co-presenting the RGD and KRSR motifs (Figure 1B), as a molecular-based strategy to improve the response of osteoblastic cells on titanium surfaces.

## 2. Results and Discussion

### 2.1. Design and Synthesis of the Molecular Biointerface

**Figure 2:**
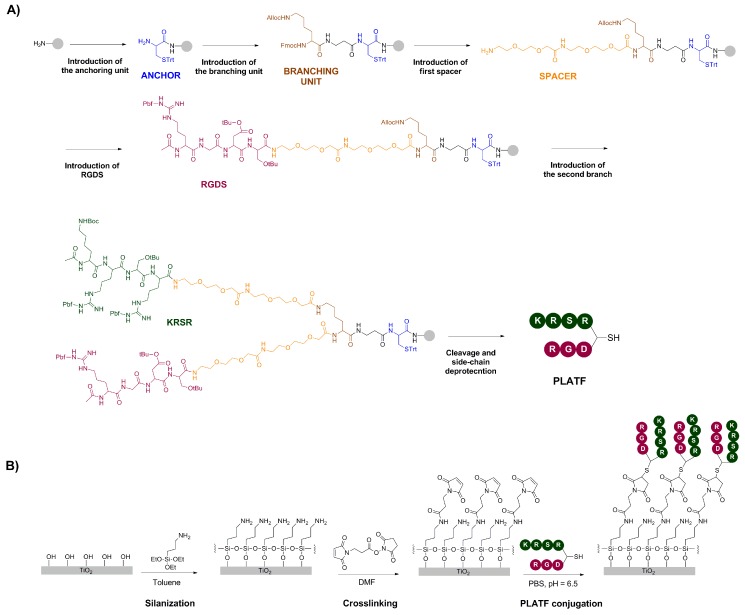
(**A**) Summary of key synthetic steps to construct the biomolecular platform (PLATF). The major features of the coating are highlighted in color; the anchor (blue), branching (brown) and spacer (orange) units, and the two bioactive peptides, KRSR (green) and RGDS (violet). Synthetic details are described in the Methods section and in the recent literature [30,33]. (**B**) Grafting of the molecules (PLATF and controls) on titanium substrates was achieved by silanization with APTES, crosslinking with a maleimide-containing molecule and subsequent addition of thiolated molecules in an aqueous buffer.

The synthesis of the biomolecular PLATF and the strategy of surface functionalization are summarized in Figure 2. The branched architecture was manually prepared, stepwise, following solid-phase peptide synthesis (SPPS) protocols (Figure 2A). SPPS is a versatile and robust method to build up modular and multifunctional constructs. The first step was the introduction of a cysteine residue (Cys, labelled blue) as an anchoring unit. This amino acid contains a thiol group as a side chain, which can be used to react with maleimide groups present at the surface via a Michael-type addition (Figure 2B). The higher nucleophilic character of thiols versus other functional groups ensures the binding of this molecule specifically at this position, thus avoiding unspecific reactions with other side chains of the molecule, which would be deleterious for the biological activity. The second step implied the coupling of an orthogonally protected (Fmoc/Alloc) lysine (Lys, labelled brown) that acts as a branching point. Indeed, selective removal of the Fmoc group with basic treatments does not affect the Alloc group, and allows growing the molecule only at the α-position. Thus, in a subsequent step, two short polyethylene glycol (PEG) chains (labelled orange) and the RGDS motif (labelled violet) were added. The PEG linker works as spacer, providing an adequate separation of the bioactive sequence (i.e., RGDS) from the surface, a crucial requirement for an optimal accessibility of the peptide to cell receptors [16,35,36,37]. The last steps involved the elimination of the Alloc group with palladium, and the construction of the second branch, consisting of another PEG spacer and the KRSR sequence (labelled green). Finally, the molecule (PLATF) was cleaved from the solid support with simultaneous deprotection of all side chains, purified and characterized as detailed in the Methods section. Grafting of the PLATF and the control linear peptides (RGD and KRSR) was carried out using aminosilanization, crosslinking with a maleimide linker and a Michael-type addition of the thiolated molecules under a slightly acidic pH (Figure 2B). This strategy of conjugation is based on a well-established method [38,39], further optimized in our group over the last years [32,33,40].

In summary, such approach yielded molecular biointerfaces reproducing integrin and proteoglycan binding regions of the ECM, by combining the sequences KRSR and RGD at a chemically defined 1:1 ratio on medically relevant titanium surfaces.

### 2.2. Physicochemical characterization of the biointerfaces

**Figure 3:**
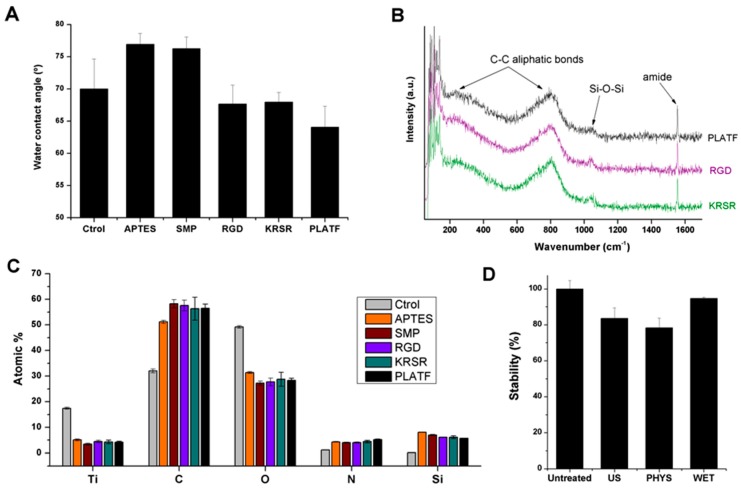
Characterization of the molecular biointerfaces. (**A**) Water contact angle (°) at each step of the functionalization. (**B**) Raman spectra (intensity in arbitrary units, a.u., as a function of wavenumbers, cm^−1^) of the bioactive surfaces. Most representative peak assignments are indicated with arrows and labels. No bands were detected for control titanium. (**C**) XPS analysis (atomic %) of relevant elements (Ti 2p, C 1s, O 1s, N 1s and Si 2p) at each step of the functionalization. (**D**) Stability of the coatings (as %) under ultrasonication (US), physiological conditions (PHYS) and wet storage (WET), as measured by XPS. The N 1s signal was analyzed for each set of samples and referenced to untreated samples (100% of stability).

The physicochemical characterization and stability of the biofunctional samples are shown in Figure 3.

The hydrophilicity of the samples was studied by measuring the contact angle of water on the functionalized surfaces (Figure 3A). Wettability is known to be an important parameter in determining protein adsorption and cell adhesion on biomaterials [41]; moreover, monitoring changes in such parameter is a useful indicator of biomolecule grafting. Indeed, silanization with APTES slightly reduced the wetting capacity of control titanium (θ [Ctrol] 70.0 ± 4.7° vs. θ [APTES] 77.0 ± 1.7°), while peptide conjugation had an opposite effect (θ [RGD] 67.7 ± 3.0°, θ [KRSR] 68.0 ± 1.5°, [PLATF] 64.1 ± 3.2°). These variations in wettability were expected and correlate well with the hydrophilicity/hydrophobicity of the coating molecules [33,34]. 

The successful grafting of the molecules was further evaluated by Raman spectroscopy (Figure 3B). In particular, Raman spectra displayed representative bands corresponding to the biomolecules. These included C-C aliphatic chains signals (e.g., broad band at 790–810 cm^−1^), a weak but detectable minor band at 1040 cm^−1^ corresponding to siloxane bonds (Si-O-Si), and of note, a strong band at 1555 cm^−1^ attributed to the peptide’s amide groups [42,43]. Interestingly, for each condition, the samples were analyzed at 4 randomly distributed different positions, obtaining in all cases very similar spectra and thus indicating a homogenous distribution of the molecules on the surfaces.

Furthermore, the chemical composition of the samples (atomic percentage of selected elements) was calculated at each step of the functionalization protocol by means of X-ray photoelectron spectroscopy (XPS, Figure 3C). Overall, silanization, crosslinking and biomolecule attachment represented an increase in the concentration of C 1s and N 1s, present in the organic molecules, and a decrease in Ti 2p and O 1s, in accordance with the formation of a sub-nanometric biointerface that masks the observable signal of titanium dioxide (TiO_2_) [33]. It is also interesting to study the evolution of Si 2p through the grafting process. This element, only present as traces in Ctrol samples (0.20 ± 0.11%), reaches a significant value upon treatment with APTES (8.04 ± 0.04%) proving the efficiency of silanization. However, the Si 2p signal is successively reduced by the addition of the crosslinker (SMP, 7.02 ± 0.21%) and the biomolecules (RGD, 6.15 ± 0.06%; KRSR, 6.16 ± 0.54%; and PLATF, 5.75 ± 0.08%), which represents further evidence of biomolecule attachment [33,40]. Interestingly, the PLATF yielded the most pronounced reduction of Si 2p concentration, in accordance with its higher molecular weight and N 1s signals (RGD, 4.06 ± 0.23%; KRSR, 4.43 ± 0.51%; and PLATF, 5.23 ± 0.25%).

XPS also proved useful to study whether the coatings produced were stable with respect to diverse challenges that could be encountered during sample manipulation (Figure 3D). More specifically, we were interested in testing the stability of the biointerfaces against ultrasonication (US), physiological conditions (PHYS) and prolonged wet storage (WET). To this end, the N 1s signal was quantified after each treatment and compared to control untreated samples. Of note, a harsh ultrasonic cleaning for a rather prolonged period (2 h) yielded only a minor detachment of the coating, which translated into an 84 ± 6% of stability. Similarly, the coatings were stable enough (78 ± 5%) to agitation in a saline buffer at 40 °C for 1 day, a condition that mimics a biological scenario. Finally, a remarkable stability (95 ± 1%) was observed when the samples were kept under agitation in an aqueous buffer for 6 days, indicating the suitability of these coatings for long-term wet storage.

### 2.3. Biological Characterization of the Biointerfaces

**Figure 4:**
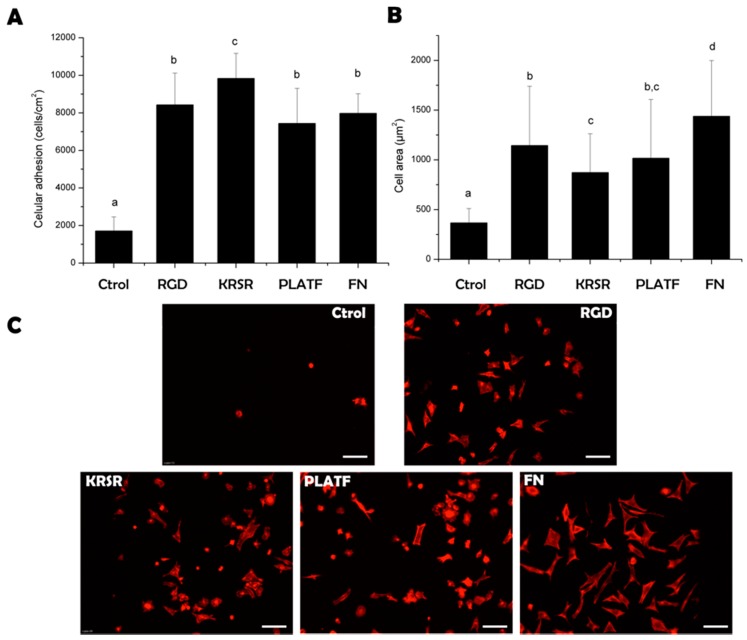
Saos-2 cell adhesion on biofunctionalized titanium surfaces after 4 h of incubation. (**A**) Cell attachment (cells/cm^2^). (**B**) Cell spreading (averaged cell area, μm^2^). Cell numbers and spreading were analyzed by immunostaining and fluorescence microscopy. (**C**) Visualization of actin filaments with TRITC-conjugated phalloidin staining (scale bar = 100 µm). Distinct letters denote statistically significant differences (*p* < 0.05) between groups.

**Figure 5:**
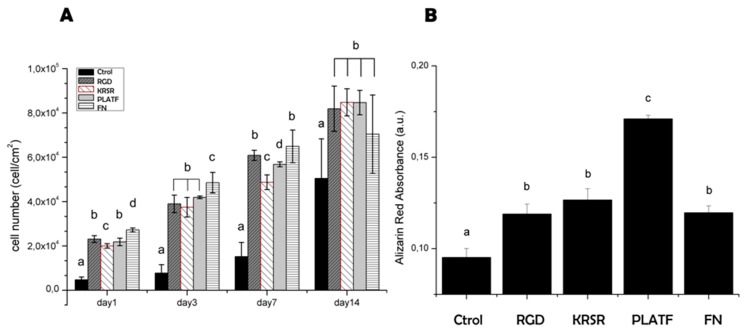
Saos-2 cell proliferation and mineralization on biofunctionalized titanium surfaces. (**A**) Cell proliferation (cells/cm^2^) after 1, 3, 7 and 14 days of culture. Quantification of cell numbers was done with Alamar Blue assay. (**B**) Quantification of calcium production by Saos-2 cells after 21 days of incubation. Staining of calcium deposits was done using ARS. Distinct letters denote statistically significant differences (*p* < 0.05) between groups.

To test the biological performance of the biomolecular coatings, osteoblast-like (Saos-2 cells) behavior was analyzed focusing on short-term (cell adhesion, Figure 4) and long-term (proliferation and mineralization, Figure 5) events.

Biomolecule grafting statistically (*p* < 0.05) improved the number of adherent cells to titanium, reaching comparable levels to samples coated with the positive control, fibronectin (Figure 4A). Of note, the heparin-binding peptide KRSR supported higher Saos-2 adhesion than the integrin-binding sequence RGD, in agreement with previous reports [18,29] and thus highlighting the high affinity of this motif for osteoblasts. However, combining RGD and KRSR within the bifunctional PLATF did not show a synergistic effect. This lack of cooperativity to enhance cell attachment was previously observed too [26,29] and may indicate that a 1:1 co-presentation of the motifs does not influence early osteoblast adhesion. This result does not correlate with the seminal work of Dee et al. [18]; however, the fact that the authors did not report the exact concentration of the mixture of peptidic ligands on the surfaces does not allow a direct comparison between the studies. Cell spreading, in contrast, followed an opposite trend (Figure 4B,C) and cells adhering on RGD-coated samples displayed a higher cell area than those on KRSR-coated ones (*p* < 0.05). This observation indicates a more prominent role of integrins over proteoglycan signaling to stimulate osteoblast spreading [31]. Accordingly, combining the two motifs (PLATF) did not enhance the values achieved by RGD alone. Yet, all peptides significantly (*p* < 0.05) improved cell spreading in comparison to plain titanium, showing well-defined cytoskeletal organizations.

The capacity of adherent osteoblasts to proliferate on the biointerfaces was analyzed after further incubating the cells for 1, 3, 7 and 14 days (Figure 5A). Overall, the bioactive sequences improved cell growth compared to control titanium at all time points, but differences within the peptides were rather modest. During the first week, a higher tendency towards increased cell numbers was observed for both RGD and PLATF in comparison to KRSR (statistical differences were found at days 1 and 7, *p* < 0.05); however, the same values of proliferation (*p* < 0.05) were detected at 14 days for all the biofunctionalized conditions. These results seem to indicate a positive role for both integrins and cell-membrane proteoglycans in supporting osteoblast proliferation, but do not demonstrate any crosstalk or cooperative effects between them. On the contrary, the extent of mineralization was significantly increased (*p* < 0.05) by the PLATF biointerface in comparison with controls (Ti, RGD, KRSR and FN). Of note, matrix mineralization is considered a relevant late marker of osteoblastic differentiation and a good indicator of bone formation in vitro. KRSR was previously shown to promote the expression of osteogenic genes on osteoblasts (including alkaline phosphatase (ALP), runx2, osteopontin and osteocalcin, among others) [23] and enhanced ALP activity and mineralization of bone marrow stromal cells [44]. RGD is also known to foster osteodifferentiation [10,32,33,45]. However, our data show for the first time that combining KRSR with RGD in a defined 1:1 ratio has a positive synergistic effect in producing calcified matrix. Taken together, these results demonstrate the potential of developing biomolecular dual coatings to improve the bioactivity of metallic implants.

## 3. Materials and Methods

### 3.1. Production of Biomolecular Interfaces

#### 3.1.1. Synthesis of Biomolecular Coatings

The dual biomolecular platform (**PLATF**, [(Ac-Arg-Gly-Asp-Ser-PEG) (Ac-Lys-Arg-Ser-Arg-PEG)]-Lys-βAla-Cys-NH_2_), and the control linear peptides, **RGD** (MPA-PEG-Arg-Gly-Asp-Ser-NH_2_) and **KRSR** (MPA-PEG-Lys-Arg-Ser-Arg-NH_2_) (PEG: 2 units of 8-amino-3,6-dioxaoctanoic acid; MPA: 3-mercaptopropionic acid) were manually synthesized on solid-phase using Fmoc-Rink amide MBHA resin (200 mg, 0.45 mmol/g) as solid support, and following synthetic protocols detailed in previous reports [30,33]. All peptides were purified by semi-preparative high-performance liquid chromatography (HPLC) and were characterized by analytical HPLC and mass spectrometry (matrix-assisted laser desorption ionization—time-of-flight, MALDI-TOF) (Table 1).

#### 3.1.2. Production of Titanium Samples

Commercially pure (c.p.) grade 2 titanium disks were cut from bars (Technalloy S.A., Sant Cugat del Vallès, Spain) and polished to a mirror-like texture (*R*_a_ below 40 nm) sequentially using SiC grinding papers and alumina suspensions (particles sizes of 1 µm and 0.05 µm) on cotton clothes. Prior to biomolecule functionalization, the surfaces were ultrasonically rinsed in cyclohexane, isopropanol, water, ethanol and acetone, and passivated with 65% (*v*/*v*) HNO_3_ for 1 h at room temperature (RT). Samples were stored N_2_-dried.

#### 3.1.3. Biomolecule Grafting

Passivated titanium samples were silanized with (3-aminopropyl)triethoxysilane (APTES, Sigma-Aldrich, St. Louis, MO, USA) according to the method developed by Xiao et al. [38,39], and further optimized in our laboratories [32,33,40]. In brief, silanization was achieved by treating the samples with 2% (*v*/*v*) APTES under an anhydrous atmosphere (nitrogen) for 1 h at 70 °C, followed by copious washes and a thermal treatment to cure the silane layer (5 min at 120 °C). Next, the aminosilanes were reacted with 2 mg/mL of *N*-succinimidyl-3-maleimidopropionate (SMP) (Alfa Aesar, Karlsruhe, Germany) in DMF for 1 h at RT. The introduction of maleimide groups assisted the grafting of the thiolated peptides, which were dissolved at 100 µM in PBS (pH 6.5) and deposited onto the samples (100 µL/disk) overnight at RT.

### 3.2. Physicochemical Characterization

#### 3.2.1. Contact Angle Measurements

Static water contact angle measurements were carried out using a Contact Angle System (OCA15 plus, DataPhysics, Filderstadt, Germany) and distilled water (1 µL-drop) as wetting liquid at RT. Contact angle values were obtained using Laplace–Young fitting and SCA 20 software (DataPhysics Instruments, Filderstadt, Germany). Three drops were analyzed per sample, and each condition by triplicate.

#### 3.2.2. Raman Spectroscopy

Spectra were recorded using a confocal Raman microscope (model inVia Qontor, Renishaw, Gloucestershire, UK) using a long focal distance ×50 objective. Measurements were done using a 532 m laser (potency 10 mW), 10 s of exposure time and 4 accumulations. To map the surfaces, each condition was analyzed at 4 random positions.

#### 3.2.3. X-ray Photoelectron Spectroscopy (XPS)

The chemical composition (atomic %) of the biomolecular coatings was studied by XPS using a non-monochromatic Mg anode X50 source (150 W) and a Phoibos 150 MCD-9 detector (D8 advance, SPECS Surface Nano Analysis GmbH, Berlin, Germany). Detector pass energy was fixed at 25 eV with 0.1 eV steps to record high resolution spectra at a pressure below 7.5 × 10^−9^ mbar. The elements of the high resolution analysis were C1s, O1s, N1s, Ti2p and Si2p, with binding energies referenced to a fixed C1s signal (284.8 eV). The spectra were analyzed using Casa XPS software (Version 2.3.19, Casa Software Ltd., Teignmouth, UK). 

#### 3.2.4. Stability Treatments

To conduct the treatments of stability, samples were first functionalized with a model linear RGD peptide, following the methods described in Section 3.1. Next, peptide-coated samples were challenged against either (i) ultrasonication in water for 2 h; (ii) agitation in PBS at 40 °C for 12 h; or (iii) agitation in water for 1 week at RT. The extent of peptide coating loss was quantified by XPS. To this end, N 1s signals were recorded, and these values were referenced to functionalized samples that were not subjected to stability treatments. Data were presented as % of stability, with untreated samples representing 100% of stability. 

### 3.3. Biological Characterization

#### 3.3.1. Cell Culture

Human sarcoma osteogenic (Saos-2) cells (ATCC, USA) were cultured in Mc Coy’s 5A medium supplemented with 10% (*v*/*v*) fetal bovine serum (FBS), 2% (*v*/*v*) 4-(2-hydroxyethyl)-1-piperazineethanesulfonic acid (HEPES), 1% (*w*/*v*) sodium pyruvate, 50 μg/mL streptomycin, 50 U/mL penicillin, and 1% (*w*/*v*) L-glutamine. Cells were incubated at 37 °C in a humidified atmosphere containing 5% (*v*/*v*) CO_2_ and the culture medium was changed twice a week. Confluent cells were detached by trypsin-EDTA and subcultured into a new flask. The experiments were performed with cells between passages 25−35. All reagents were purchased from Sigma−Aldrich, unless otherwise noted.

#### 3.3.2. Immunofluorescence Analysis: Cell Adhesion and Spreading

The capability of the different coated surfaces to support cell adhesion and spreading was evaluated by means of immunofluorescence staining. To this end, prior to cell seeding, the surfaces were rinsed in PBS and blocked with bovine serum albumin (BSA, 1% (*w*/*v*), 50 min) to reduce non-specific cell attachment. Subsequently, cells were seeded at 5 × 10^4^ cells/mL (25,000 cells per disk) in serum free medium and incubated at 37 °C. Cells were allowed to attach for 4 h. After this time, cells were fixed with paraformaldehyde (PFA, 4% *w*/*v* in PBS, 20 min), permeabilized with Triton X-100 in PBS (0.05% (*w*/*v*), 20 min) and blocked with BSA in PBS (1% (*w*/*v*), 30 min). TRITC-conjugated phalloidin (1:300, in permeabilizing buffer) was used to stain actin fibers (1 h) and 4′,6-diamidino-2-phenylindole (DAPI) (1:1000, in PBS-glycine 20 mM) was used for nuclei (2 min), both in the dark. Between all steps, samples were rinsed three times with PBS-glycine (5 min). Ti disks were mounted and examined under a fluorescence inverted microscope (AF7000, Leica, Wetzlar, Germany), and images were processed using the Fiji/Image-J package. The number of adhered cells was assessed by counting DAPI-stained nuclei and cell spreading by measuring the cell area. 

#### 3.3.3. Cell Proliferation and Mineralization

For long-term analysis of cellular behavior, samples were washed and blocked as described for the immunofluorescence experiments. Next, Saos-2 cells were plated at a concentration of 2 × 10^4^ cells/mL (10,000 cells per disk) in serum-free medium and incubated for 4 h. After this time, the medium was aspired and FBS-supplemented medium was added. For proliferation studies, on days 1, 3, 7, and 14, the medium was replaced with Alamar Blue (AB)-containing medium (10% (*v*/*v*), ThermoFisher, Merelbeke, Belgium) for 3 h and the fluorescence of the dye quantified using a microplate reader (λ_ex_ = 560 nm; λ_em_ = 590 nm). To obtain cell number from the fluorescence read-out, a standard curve of defined cell concentrations was applied. Additionally, to study the mineralization process, the extracellular calcium deposits produced by osteoblast-like cells were stained using Alizarin Red S (ARS, Sigma-Aldrich). In this case, cells were incubated for 21 days in osteogenic medium, i.e., medium supplemented with 10 mM β-glycerophosphate, 50 μg/mL ascorbic acid and 100 nM dexamethasone, and fixed with 4% (*w*/*v*) PFA. Ti disks were then washed twice with Milli-Q water and incubated under orbital shaking for 20 min with 500 μL/disk of 40 mM ARS (pH 4.2). Copious washings with Milli-Q water were applied to eliminate the excess dye. Finally, to elute the stain, the samples were treated with 300 μL/disk of cetylpyridinium chloride (CPC) buffer (10% (*w*/*v*) in 10 mM NaH_2_PO_4_, pH 7) for 30 min. To quantify the dye, the supernatant was then collected, diluted 1:2 with CPC buffer and 100 μL aliquots were plated to measure the absorbance at 570 nm.

### 3.4. Statistical Analysis

If not stated otherwise, all samples were analyzed by triplicate. In addition, cellular assays were repeated at least in two independent assays. All data are expressed as average values (X) ± standard deviation (SD). Statistically significant differences between groups were assessed by 1-way ANOVA followed by post hoc pairwise comparisons using the Tamhanne and Scheffe post hoc test, depending on the homogeneity of the variance. Differences were also analyzed by the nonparametric Kruksall−Wallis test. The software used for statistical analysis was SPSS statistics (IBM, Armonk, NY, USA).

## 4. Conclusions

Ideally, biomolecule-based coatings designed to functionalize metallic implants should have the capacity to enhance the adhesion of bone forming cells and improve their differentiation. This may be achieved by mimicking bone ECM signaling using peptidic mixtures, but current approaches often fail to control the exact amount and ratio of peptidic motifs exposed on the surface. In this work, we reproduced integrin and proteoglycan binding by combining RGD and KRSR sequences at a defined 1:1 ratio within a dual molecular biointerface. This strategy statistically improved the number, area, proliferation and mineralization of osteoblasts compared to control titanium. The co-presentation of RGD and KRSR using this method did not show a synergistic effect on cell adhesion or proliferation in comparison with the linear controls; however, it did improve the production of a calcified matrix by osteoblasts in a cooperative manner. Mineralization is considered an important late marker of osteodifferentiation, thus the dual biointerfaces generated may prove useful to bioactivate metallic implants in clinical settings. Further studies in this direction are warranted. 

## Figures and Tables

**Table 1:** Characterization of the biomolecules.

Code	*t*_R_ (min) ^a^	Purity (%) ^a^	*m*/*z* calcd.	[M + H]^+^
PLATF	4.253	97	1926.98	1927.13
RGD	5.790	>99	810.35	811.49
KRSR	5.348	>99	922.50	923.52

^a^ Retention times (*t*_R_) and purity (%) calculated by HPLC using a reversed-phase XBridge BEH130 C-18 column (4.6 mm × 100 mm, 3.5 µm) (Waters, Milford, MA, USA) and a photodiode array detector (Waters 2998). Linear gradients were run at a flow rate of 1.0 mL/min over 8 min at room temperature: PLATF (5 to 40% ACN); RGD (20 to 60% ACN); KRSR (0 to 40% ACN).
